# A State of the Art Review on the Recalcitrant Candidiasis as a Clue of Inborn Errors of Immunity in Newborns

**DOI:** 10.1155/jimr/9308118

**Published:** 2026-07-07

**Authors:** Mahsa Fattahi, Pegah Tamimi, Aliasghar Ghaderi, Mohammad Reza Fazlollahi, Zahra Pourpak

**Affiliations:** ^1^ Immunology, Asthma and Allergy Research Institute, Tehran University of Medical Sciences, Tehran, Iran, tums.ac.ir; ^2^ Children’s Medical Center, Pediatrics Center of Excellence, Tehran University of Medical Sciences, Tehran, Iran, tums.ac.ir; ^3^ Department of Medicine, Medical School of Tehran University of Medical Sciences, Tehran, Iran

**Keywords:** candidiasis, KREC, newborn screening, primary immunodeficiency diseases, recalcitrant candidiasis, TREC

## Abstract

**Background:**

Defects in the development or function of the immune system are characteristic of inborn errors of immunity (IEI), a diverse group of inherited disorders. IEI can manifest in various ways during the neonatal stage, posing a challenge in their identification and treatment. The family who have a background of IEI, including a previous instance of an infant’s death caused by infection, sepsis from bacteria in children requiring intravenous (IV) antibiotics, and failure to thrive, as well as newborns with fungus infection such as chronic recalcitrant candidiasis. This review sought to clarify the significance of recalcitrant candidiasis in identifying IEI in newborns. To enhance healthcare strategies, we make it a priority to keep data on managing difficult candidiasis up‐to‐date and consider the potential benefits of implementing these newborn screening programs for IEI.

**Methods:**

In this review, we highlight the state‐of‐the‐art recalcitrant candidiasis‐based diagnostic strategies of the IEI in newborns. The article is organized as follows: In Section 2, different clinical features of immunodeficiencies in newborns will be summarized, with emphasis different pathogens are associated in different types of immunodeficiencies. Also, different types of candidiasis which are related to each type of IEI will be presented in Section 3. The challenges diagnostic tests to identify of IEI (screening and definite diagnosis) and the type of diagnostic tests for confirmation of recalcitrant candidiasis from bench to bedside will be covered in Sections 4–6. The concept of a patient’s treatment who has recalcitrant candidiasis will be addressed along with this paper in Section 7. Implication for practice will be discussed in Section 8.

**Results:**

The most previous study shows antifungal treatment failure, caused by resistant or tolerant activity toward commonly used antifungal medications, is a probable cause for the presence of recalcitrant candidiasis in newborns. It is important for clinicians to consider the possibility of underlying conditions and order specific tests to identify the type of Candida and determine the best treatment for newborns with persistent candidiasis.

**Conclusions:**

To sum up, remember to consider immunodeficiency when deciphering abnormal treatment response for candidiasis. Resistant or dose‐dependent candidiasis lab results in a neonate may signal a potential immunodeficiency, leading to more medical and lab examinations to confirm or rule it out. It can be said that the recalcitrant candidiasis may be considered as a clue about the nature of the underlying immune deficiency. A comprehensive knowledge of recalcitrant candidiasis is imperative in identifying IEI, underscoring the crucial role of well‐trained local pediatricians in early detection. The importance of implementing an early detection program for IEI couldn’t be overstated, as it has been proven to significantly enhance quality of life and life expectancy by allowing for timely medical interventions in cases of IEI and significantly reduces medical expenses.

**Summary**

Recalcitrant candidiasis may hold clues about the nature of the underlying immune deficiency. This would be helpful in the early detection of inborn errors of immunity (IEI) in Newborns.

## 1. Introduction

Following birth, neonates, whether premature or full‐term, undergo profound immunological changes to adapt to extrauterine life [1–3]. Genetic mutations affecting this immune development can lead to primary immunodeficiency diseases (PIDs) or inborn errors of immunity (IEI), rendering affected individuals susceptible to infections, autoimmunity, allergies, chronic inflammation, and malignancies [4, 5].

Although historically rare, IEIs are now more prevalent, with an estimated occurrence of 1 in 2000 live births [6], affecting 6–8 million individuals globally, with prevalence figures in symptomatic patients ranging from 1:8500 to 1:100,000 depending on the registry [7–9]. In the United States, the estimated prevalence of diagnosed IEI is ~1 in 1200 individuals [10].

The classification of IEIs has expanded to encompass 555 distinct disorders across 10 categories, including combined immunodeficiencies (CIDs), antibody deficiencies, immune dysregulation, and phagocytic dysfunction [5]. Early diagnosis of immunodeficiency disorders in newborns remains particularly challenging due to the nonspecific nature of early clinical manifestations and the immaturity of the immune system. In this age group, recalcitrant candidiasis, especially when unresponsive to standard antifungal therapy, should be considered an important warning sign of IEIs in newborns. In newborns with immunodeficiency, especially severe CID (SCID), if a hematopoietic stem cell transplant is performed within the first month of life, the survival rate is around 90%, and this chance gradually decreases overtime.

This review aims to highlight the clinical relevance of recalcitrant candidiasis as a sentinel sign of IEI in neonates. Furthermore, it outlines the latest diagnostic approaches and proposes the integration of newborn screening programs to facilitate the early identification and intervention for IEI.

To achieve this, the review is structured as follows:

Section 2: Overview on Clinical Feature of Inborn Errors of Immunity in Newborns
Section 3: Overview on *Candida* Infection in Newborn With IEI
Section 4: Initial Clinical Assessment
Section 5: How We Confirm Recalcitrant Candidiasis in Newborns With IEI
Section 6: The Appropriate Diagnostic Tests for IEIs Screening in Newborns With Recalcitrant Candidiasis
Section 7: Treatment (Current Therapeutic Strategies, With Emphasis on Antifungal Regimens and Immunologic Interventions in Neonates)
Section 8: Implications for Practice

## 2. Overview on Clinical Feature of Inborn Errors of Immunity in Newborns

Diagnosing IEI in newborns is challenging due to the difficulty in assessing their immune systems, which may mask underlying deficiencies. Early diagnosis is essential for initiating appropriate therapy to prevent infections and minimize tissue damage. Diagnosing IEI can manifest in numerous ways, leading to visits to various healthcare providers. Clinicians should be alert to the possibility of an immune deficiency when certain clinical signs are persistent. Key symptoms include multiple ear or sinus infections, chronic pneumonia, inadequate response to antibiotics, failure to thrive, persistent fungal infections, recurrent abscesses, and a family history of IEI [11] (http://www.info4pi.org).

**Figure 1:**
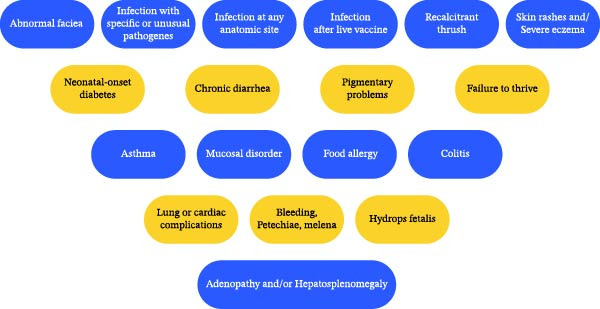
Clinical features of immunodeficiencies in newborns [7, 12, 13].

Red flags that appear in the early months of life, whether individually or together, should prompt an investigation into potential immune deficiency (Figure 1) [12].

Different pathogens are associated with different types of immunodeficiencies, for instance, recurrent sinopulmonary infections with encapsulated bacteria are often seen in B‐cell disorders, while *Pneumocystis jirovecii* pneumonia is linked to T cell deficiencies, such as SCID [12, 14–16] and CD40 ligand deficiency [16]; severe phagocytic, humoral, or T cell deficiencies may present with infections from *Pseudomonas aeruginosa* [12]. *Staphylococcus aureus* infections suggest phagocyte dysfunction [12]; recurrent *Staphylococcal* skin infections [17], abscesses, lung cysts, or pneumonia were linked to hyper‐IgE syndrome [17].

Viral infections such as *Herpes simplex* virus (HSV), *Cytomegalo* virus (CMV), and *Epstein–Barr* virus (EBV) are more frequent in NK cell or combined T cell defects [18–21]. Patients with severe immunodeficiencies like SCID and X‐linked agammaglobulinemia (XLA) may also experience infections from live vaccines, such as Bacillus Calmette‐Guerin (BCG) or oral polio [12]. While complement deficiencies predispose individuals to *Neisserial* infections [22]. Infections with mycobacteria are linked to defects in interferon gamma (IFN‐γ) or interleukin (IL) receptors [12, 23]. Persistent warts and fungal infections are key signs of immune system dysfunction. For example, recurrent *Molluscum contagiosum* or persistent, extensive warts are commonly seen in T cell defects, innate immune deficiencies, or the combined defect known as warts, hypogammaglobulinemia, infection, and Myelokathexis syndrome (WHIM) [12]. Additionally, fungal infections such as cryptococcosis, histoplasmosis, and *Talaromyces* species infections are noted in IEI patients [24]. Notably, *Candida parapsilosis* fungemia was observed in a 17‐day‐old neonate diagnosed with SCID [7, 25].

As mentioned above, the presence of specific organisms such as recalcitrant candidiasis infection should be cause for concern and does not mean there is a lab mistake or insignificant finding; IEI should be taken into account [7].

The literature on invasive and mucocutaneous *Candida* infections in IEI was analyzed in this study through different subjects.

## 3. Overview on *Candida* Infection in Newborn With IEI

Newborns are highly susceptible to *Candida* infections, especially during the neonatal period, when their immune systems are still developing. While newborns usually have minimal fungal colonization at birth, they often acquire it soon after, either through vertical transmission from the mother or horizontal exposure in the hospital environment. Increased risks for candidiasis in neonates include low gestational age, low birth weight, the use of broad‐spectrum antibiotics, central venous catheters, intravenous (IV) feeding, previous *Candida* infections, extended urinary catheter use, and known impaired T‐cell immunity or positive familial history of any IEI [26]. When evaluating potential IEI in neonates with candidiasis, it is critical first to exclude secondary causes related to the above risk factors. In patients with IEI, intrinsic immune defects increase susceptibility to recurrent, severe, or unusual infections, often involving the skin and mucous membranes, which is driven by a complex interplay of failed immune clearance, pathogen evasion strategies, and host genetic susceptibility [27, 28]. *Candida* infections have been reported across multiple IEI types [29–32]. Key pathophysiological immune pathways involved include mutations in critical genes involved in immune signaling and response, impaired neutrophil function, defective pattern recognition receptors (PRRs), and complement evasion [28]. Key genes involved include all genes related to chronic mucocutaneous candidiasis (CMC): *STAT1*, *IL17RA/IL17RC, DOCK8*, *CARD9*, *TLR-3*, and *AIRE*, and also all genes related to SCID, all of which impair the immune system’s ability to effectively combat fungal infections. Identifying these genetic defects can lead to earlier diagnosis and targeted therapies for affected individuals [28].

**Figure 2:**
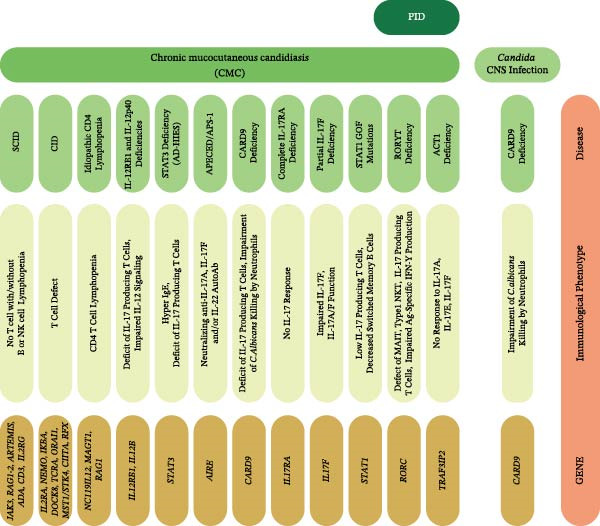
Relationship between various forms of candidiasis and specific IEI subtypes [35].

In newborns, protection against *Candida* relies predominantly on mucosal immunity because adaptive immune responses are functionally immature. An intact epithelial barrier with tight junctions and mucus limits fungal adherence and invasion, while antimicrobial peptides such as defensins, lysozyme, and lactoferrin directly inhibit fungal growth. Recognition of *Candida* by PRRs induces cytokines that promote Th17 differentiation and IL‐17 production, which in turn stimulates epithelial cells to produce antimicrobial peptides and recruit neutrophils. Maternal secretory IgA from breast milk further prevents fungal adhesion without provoking excessive inflammation, and a balanced microbiota competitively restricts fungal overgrowth. Recalcitrant candidiasis develops when this integrated mucosal defense network is disrupted, particularly when the IL‐17/Th17 axis is impaired, leading to reduced antimicrobial peptide production, defective neutrophil recruitment, weakened epithelial containment, and persistent fungal colonization. Thus, chronic or recalcitrant candidiasis in neonates serves as a clinical indicator of dysfunction within the mucosal immune axis and may suggest an underlying IEIs [33, 34]. The correlation between various types of candidiasis and specific subtypes of immune deficiencies (IEI) is depicted in Figure 2.

### 3.1. Recalcitrant CMC and Recalcitrant Thrush

A definite recalcitrant or refractory infection refers to an infection that is persistent and resistant to treatment despite appropriate and prolonged therapy. In the context of fungal infections like candidiasis, this means that the infection does not resolve with standard antifungal treatments and may require alternative or more aggressive therapeutic approaches. *Candida* spp. They can develop resistance to antifungal agents through multiple mechanisms. Resistance to azole drugs, such as fluconazole, may occur via upregulation of efflux pumps (e.g., *CDR*1, *CDR*2, and *MDR*1), mutations in the *ERG*11 gene affecting the target enzyme lanosterol 14α‐demethylase, or biofilm formation that reduces drug penetration. Resistance to other FDA‐approved antifungals, including echinocandins and polyenes, can result from mutations in *FKS* genes or alterations in membrane sterol espectively. Understanding these mechanisms is critical for guiding therapy in patients with refractory candidiasis, particularly in the context of underlying immunodeficiencies [36, 37].

This type of infection is often associated with underlying immune system deficiencies, such as IEI, which impair the body’s ability to combat pathogens effectively.

CMC is characterized by persistent or recurrent *Candida* infections affecting the oral cavity, esophagus, gastrointestinal and genitourinary tracts, nails, and skin, typically caused by *Candida albicans* [38].

While mucocutaneous infections such as thrush and diaper dermatitis are common even in otherwise healthy full‐term infants [39], premature neonates are particularly prone to developing severe mucocutaneous and disseminated candidiasis [38, 40, 41].

The incidence of CMC is significantly increased among patients with IEI [27]. T cell defects, particularly impaired TH17‐mediated immunity, are central to the CMC pathogenesis. SCID [42, 43], CIDs [44], and DiGeorge syndrome all impair T cell function [45] and predispose to chronic candidiasis [46–48]. In some cases, persistent mucocutaneous candidiasis may be the first clinical manifestation of SCID in infancy [49]. CMC may present as the first symptom during infancy, particularly in individuals with SCID [50].

It is noteworthy that although CMC can present early, only a small proportion of neonates exhibit symptomatic disease in the first months of life. This includes those with familial CMC or infants with additional susceptibility factors such as extremely low birth weight (<750 g), third‐generation cephalosporin use by Day 3 of life, or delayed enteral feeding [1, 26, 51]. Therefore, CMC presentation in early infancy should raise suspicion of an underlying IEI [27].

Cases of IL‐12Rβ_1_ deficiencies, subtypes of IEI [52], were seen with recurrent thrush [53]. Persistent oral candidiasis in a neonate or young infant may, in fact, be a presenting symptom of SCID [42, 43]. Thrush was observed in more than 53% of patients with SCID [54]. Purine nucleoside phosphorylase deficiency (PNP deficiency), which is a subgroup of SCID, showed severe oral thrush [55].

Rare cases of biallelic missense mutations in ACT1 (TRAF3IP2), an adaptor molecule critical for IL‐17 receptor signaling, have been identified in siblings with recurrent oral thrush, onychomycosis, and *S. aureus* skin infections [56]. This mutation affects the encoding of an adaptor molecule that interacts with IL‐17 receptors and triggers the activation of downstream pathways, including NF‐κB [56].

### 3.2. Recalcitrant Central Nervous System (CNS) Disease

CNS disease is seen in approximately half of disseminated candidiasis cases. Even when treatment is sufficient, the mortality rate ranges from 10% to 30%, and neurological issues continue in 18%–29% of survivors [57, 58]. Survivors frequently face neurodevelopmental difficulties, underscoring the need for the diligent tracking of their neurodevelopmental markers [26, 59, 60]. CNS candidiasis is most often seen following neurosurgical procedures or in premature neonates. The diagnosis should trigger consideration of underlying immune defects such as CARD9 deficiency [61, 62]. Although CARD9 deficiency is classically diagnosed in childhood or adulthood, the insights gained from this rare PID are highly relevant to neonates, who represent a major risk group, especially for CNS candidiasis.

CARD9 is currently the only molecule proven to be nonredundant for protection against *Candida* infections of the CNS in humans. Studies of CARD9‐deficient patients have demonstrated that CARD9 is essential for neutrophil recruitment and antifungal effector function within the CNS and that its absence results in a functional “CNS neutropenia” despite normal systemic neutrophil counts. This mechanism directly explains the strong association between CARD9 deficiency and devastating *Candida* meningoencephalitis.

Importantly, CARD9‐sufficient neonates exhibit immunological features that closely resemble key aspects of CARD9 deficiency, particularly in the context of CNS antifungal immunity. Neonates have impaired neutrophil chemotaxis, reduced neutrophil effector function, and diminished recruitment of neutrophils into the cerebrospinal fluid (CSF) during CNS *Candida* infections, while responses to bacterial CNS infections remain largely intact. These defects parallel the CNS‐restricted neutrophil recruitment failure observed in CARD9‐deficient patients.

In addition, neonates have an immature innate immune system and a Th2‐skewed adaptive immune profile, with reduced Th1 and Th17 responses, immune pathways that are critical for antifungal defense. Neonatal neutrophils also display reduced expression of genes involved in IL‐1 signaling, inflammasome activation, and nitric oxide production, all of which are CARD9‐dependent pathways known to be essential for controlling fungal infections.

Therefore, although true CARD9 deficiency is rare in neonates, the pathophysiological mechanisms uncovered through CARD9‐deficient patients provide a mechanistic framework for understanding why neonates are uniquely susceptible to CNS candidiasis. These insights underscore the importance of considering CARD9‐related pathways, rather than only genetic CARD9 mutations, when assessing the risk and designing therapeutic strategies for neonatal CNS candidiasis.

Ultimately, studying CARD9 deficiency not only informs the management of patients with this rare PID but also guides the development of adjunctive immune‐based therapies aimed at enhancing neutrophil recruitment and function in CARD9‐sufficient but immunologically immature neonates, a population with high morbidity and mortality from CNS *Candida* infections [63].

Neonates with candidemia and symptoms resembling meningoencephalitis should be monitored for CNS disease. Take into consideration that the CSF results for *Candida* infection may not be entirely dependable [64, 65]. The treatment success rate for *Candida* meningitis varies based on the risk assessment. The mortality rate for patients with both HIV and *Candida* meningitis is 31%, but this rate falls to 11% for those who experience *Candidal* meningitis after a neurosurgical intervention. Infants born prematurely who have candidemia and *Candida* meningitis face a much greater risk of mortality and neurodevelopmental disabilities (60%) compared to a control group of infants (28%) [65–67]. The following clinical algorithm can help guide the diagnostic workup and management of neonates presenting with recurrent or severe candidiasis, with a focus on identifying underlying IEI.

## 4. Initial Clinical Assessment

### 4.1. Clinical History

Collect detailed history, including the age of onset, pattern of infections, and whether there is a family history of immune deficiencies.

### 4.2. Clinical Signs

Look for mucocutaneous candidiasis (oral thrush, diaper rash, and genital infections) or systemic candidiasis (fever and organ involvement).

## 5. How We Confirm Recalcitrant Candidiasis in Newborns With IEI

Identifying recalcitrant candidiasis in neonates, particularly those with IEI, is challenging and requires timely laboratory testing for accurate diagnosis and effective treatment. While direct microscopy and Sabouraud agar culture are commonly used for initial confirmation, these techniques can be hindered by factors such as slow *Candida* growth or prior antifungal treatment, leading to potential false negatives. Additionally, traditional culture methods may fail to distinguish between closely related or emerging *Candida* spp. Molecular techniques like Polymerase chain reaction (PCR) assays, which can detect pathogens within 24–48 h, are increasingly vital, especially when antifungal therapy suppresses fungal growth. For fungal DNA detection and species differentiation, ribosomal gene regions (such as 18S rDNA) and the internal Transcribed Spacers (ITS1 and ITS2) are commonly targeted. However, for species complexes like *Candida albicans* and *Candida parapsilosis*, the ITS regions may lack sufficient variability. In such cases, more variable targets, such as the *hyphal wall protein 1* (*HWP1*) gene, provide a higher resolution. PCR‐restriction fragment length polymorphism (PCR‐RFLP) assays and sequencing approaches offer further refinement in species identification.

**Figure 3:**
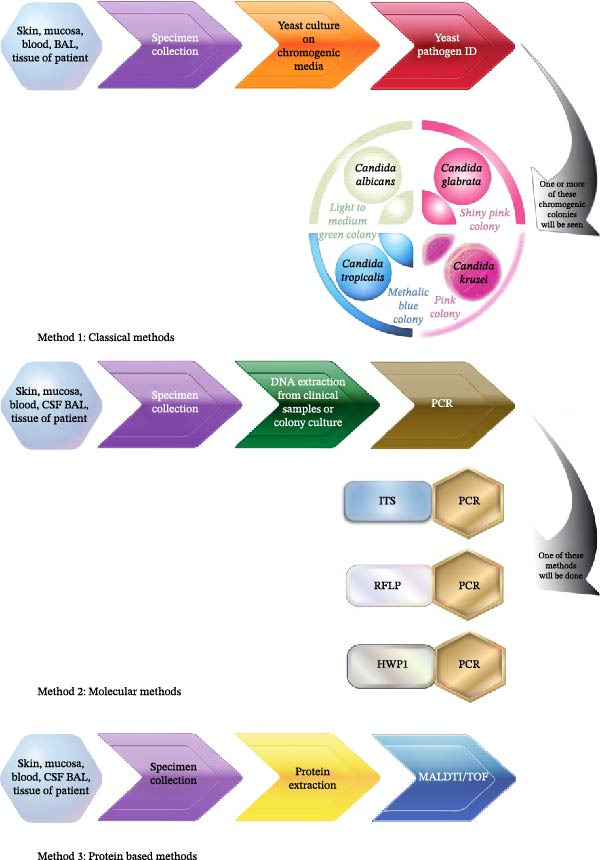
Current rapid diagnostics for species identification.

Matrix‐assisted laser desorption/ionization–time‐of‐flight mass spectrometry (MALDI‐TOF MS) has also revolutionized fungal diagnostics. This technology allows for rapid, accurate, and cost‐effective identification of *Candida* spp. based on their unique protein spectra. The major platforms include Bruker’s Microflex, bioMérieux’s VITEK MS, Shimadzu’s Axima Performance, and ASTA’s IDSys LT systems. The latest rapid diagnostic techniques for species identification are presented in Figure 3.

Clinicians must maintain a high index of suspicion for antifungal resistance, particularly recognizing intrinsic resistance in species such as *Candida krusei*, *Candida glabrata*, and the emerging *Candida auris*. While antifungal susceptibility testing (AST) provides valuable information about the *in vitro* response of pathogens, interpretation must be cautious. Minimum inhibitory Concentration (MIC) values alone do not guarantee clinical outcomes; host immune status, site, and severity of infection also play crucial roles.

**Figure 4:**
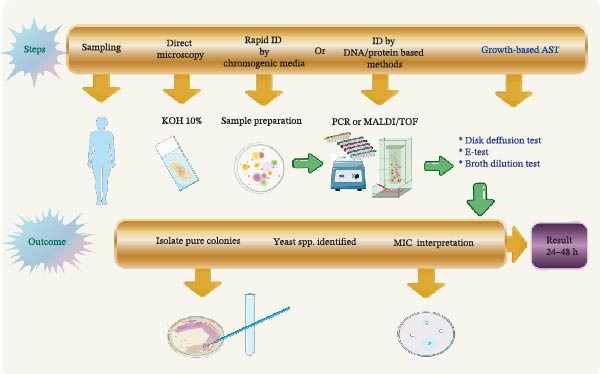
Current culture‐based method for AST, steps, and outcomes.

Although AST is particularly recommended for recurrent or persistent infections, it should be combined with a thorough immunological assessment to investigate potential underlying IEIs. Early identification of immunodeficiency is essential for optimizing patient outcomes, emphasizing the need for comprehensive clinical, microbiological, and immunological evaluations in newborns presenting with resistant candidiasis. The tests and the process are summarized in Figure 4.

## 6. The Appropriate Diagnostic Tests for IEIs Screening in Newborns With Recalcitrant Candidiasis

Antifungal treatment failure, often due to resistance or tolerance to commonly used antifungal agents, is a probable cause of recalcitrant candidiasis in newborns. In such cases, clinicians must consider the possibility of underlying immunodeficiencies. Specific diagnostic tests are essential to identify *Candida* spp. and to investigate potential IEIs, formerly referred to as PIDs, that may predispose newborns to persistent infections.

When IEI is suspected, consultation with a specialist trained in clinical immunology is crucial. Initial immunological screening should include rapid and accessible tests to assess humoral, cellular, and phagocytic immune functions. Basic immunological evaluation includes a complete blood count (CBC) and blood smear [68]. Lymphocyte subset (CD3, CD4, CD8, CD19, and CD56, respectively T4, T8, B, and NK) [69] analysis by flow cytometry, measurement of immunoglobulin levels (IgG, IgA, IgM, and IgE), examine the level and/or function of specific complement proteins, and the nitro blue tetrazolium (NBT) test, dihydrorhodamine (DHR) assay for assessment of phagocytic function, and screening for chronic granulomatous disease (CGD) [68]. If these initial investigations suggest immunological abnormalities, more advanced assessments such as T cell functional assays, detailed immune phenotyping, and targeted or whole‐genome genetic testing should be pursued in partnership with a clinical immunologist.

**Figure 5:**
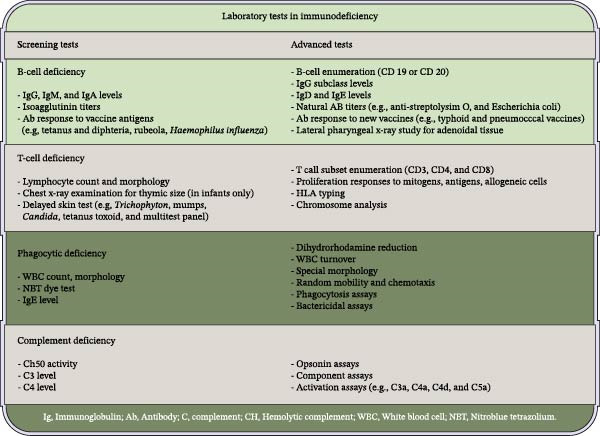
Screening test for IEI in case of clinical suspicious.

Figure 5 provides an overview of the recommended screening workflow for suspected IEI in newborns presenting with refractory candidiasis.

The importance of implementing an early detection program should be emphasized enough as it has been proven to significantly enhance quality of life and life expectancy by allowing for timely medical interventions in cases of IEI and significantly reduce medical costs.

A pivotal tool for early diagnosis is dried blood Spot (DBS) testing, which allows for immune screening within the first days of life [70]. The quantification of T cell receptor excision circles (TRECs) and kappa deleting recombination excision circles (KRECs) through DNA‐based methods was used to calculate this mean [71].

When it comes to detecting SCID [72] and XLA [73], these assays are highly sensitive with a predictive value of ~50%, and T cell lymphopenia has a high sensitivity of almost 100% in diagnosing SCID and leaky SCID [74].

SCID is one of the most severe clinical presentations of IEI. As such, it acts as a notable reference for explaining immunological examinations, and we will investigate further issues related to this condition.

Although routine SCID newborn screening is widely implemented in private healthcare systems, its adoption in public health sectors can be challenging due to the high initial costs. Nevertheless, alternative approaches, such as multiplex PCR‐based DBS analysis, can improve cost‐effectiveness at scale. Research led by the Jeffrey Modell Foundation (JMF) has developed a validated decision‐support algorithm to assist health ministries in evaluating the feasibility and economic implications of SCID newborn screening based on regional data [75, 76].

Severe CID is currently detected by a newborn screening test using the detection of TREC in some countries [77–81]

Research has proposed protein‐based assays from DBS for detecting complement deficiencies, such as C2 and C3 deficiencies, enabling preventive interventions [82]. While functional protein assays currently remain the gold standard for diagnosing complement and granulocyte disorders, emerging techniques, including whole genome sequencing (WGS), are under investigation for their potential role in future newborn screening panels for IEIs [83–85]. Conduct a next‐generation sequencing (NGS) panel for IEI. This can identify defects in key immune genes such as *STAT1*, *IL17RA/IL17RC, DOCK8*, *CARD9*, *AIRE*, and *TLR-3* [86].

## 7. Treatment (Current Therapeutic Strategies, With Emphasis on Antifungal Regimens and Immunologic Interventions in Neonates)

Over the past decade, treatment options for invasive candidiasis (IC) have expanded significantly. However, the pharmacokinetics, safety, and efficacy of antifungal agents in premature neonates remain inadequately defined [87]. Despite the use of dosing strategies based on either weight or body surface area (Table 1), weight‐based dosing is generally preferred in clinical practice due to its simplicity. Notably, there is considerable variability in reported maximum doses among antifungal agents, reflecting limited evidence in this vulnerable population [87].

**Table 1:** Dosing of antifungal agents in neonates and infants.

Dosing of antifungal agents in neonates and children [87]					
Drug	*N* (Infants)	Dose mg/kg/day	Birth weight (g)	Gestational age (week)	Comments
Amphotericin B deoxycholate	13	1	1200 (800)	27 (4.9)	N/A
Lipid amphotericin B	28	5	1060 (480–4900)	27 (24–41)	Infant weight highest predictor of clearance
Fluconazole	55	12	1020 (451–7125)	26 (23–40)	Loading dose maybe required
Micafungin	43	10–12	1162 (530–4500)	27 (23–40)	N/A
Anidulafungin	0	1.5	N/A	N/A	NA
Voriconazole	0	4 mg/kg every 12 h	N/A	N/A	N/A
Caspofungin	0	50 mg/m^2^/day	N/A	N/A	N/A

### 7.1. Treatment of CNS Candidiasis

For neonates with suspected or confirmed Central CNS candidiasis, amphotericin B remains the first‐line therapy. Both amphotericin B deoxycholate and lipid formulations have demonstrated antifungal activity with CNS penetration. In rabbit models of *Candida* meningoencephalitis, amphotericin B deoxycholate and liposomal amphotericin B showed superior efficacy compared to other formulations [88]. When selecting amphotericin B formulations, clinicians must weigh the advantages and limitations: liposomal amphotericin B achieves superior CSF concentrations but has lower urinary excretion, whereas amphotericin B deoxycholate provides higher urinary levels but lower CSF concentrations [88].

The addition of flucytosine to amphotericin B‐based regimens may enhance fungal clearance in CNS infections. Despite gastrointestinal side effects that may limit oral feeding in neonates, early combination therapy is recommended when feasible. Notably, a prospective study found a longer median time to CSF sterilization in infants treated with amphotericin B deoxycholate plus flucytosine (17.5 days) compared to amphotericin B alone (6 days) [26]. These findings highlight the need for careful clinical judgment regarding the timing and tolerability of combination therapy.

Voriconazole is generally not recommended for neonatal CNS candidiasis due to limited pharmacokinetic data. However, in cases of *Candida glabrata* or *Candida krusei* meningitis, where resistance patterns necessitate alternative therapy, voriconazole may be considered after initial treatment with amphotericin B and/or flucytosine. Therapeutic drug monitoring (TDM) is essential due to voriconazole’s variable metabolism. Posaconazole is not recommended for CNS candidiasis given its poor CSF penetration [89]. The role of echinocandins in neonatal CNS infections remains investigational. Although echinocandins achieve detectable levels in the brain parenchyma at high doses in animal models, they exhibit poor CSF penetration. Consequently, their use in CNS candidiasis is currently not supported by robust clinical evidence [89, 90]. Overall, early initiation of appropriate antifungal therapy is critical to prevent CNS invasion and improve outcomes in neonates with IC.

### 7.2. Immune‐Based Therapeutic Strategies

In neonates with underlying IEI, adjunctive immune‐based therapies may be necessary to control invasive fungal infections (IFIs). Immunotherapeutic approaches primarily focus on augmenting neutrophil numbers and function given their central role in antifungal host defense.

### 7.3. Granulocyte‐Focused Therapies

Granulocyte transfusions to temporarily boost phagocytic capacity [91]. Growth factor infusions, such as granulocyte colony‐stimulating factor (G‐CSF) and granulocyte‐macrophage colony‐stimulating factor (GM‐CSF), to enhance neutrophil proliferation [92]. Cytokine‐based augmentation, involving agents like IFN‐γ and IL‐15, to stimulate neutrophil and macrophage activity [93, 94]. However, granulocyte transfusion therapy is limited by challenges in *ex vivo* expansion, poor persistence of transfused cells due to apoptosis, and sequestration in the pulmonary vasculature [95].

Thus, these interventions remain experimental in neonates and are generally reserved for selected cases. Recent advances in immunotherapy include the adoptive transfer of pathogen‐specific T cells, dendritic cell vaccines, and the development of monoclonal antibodies or recombinant pentraxins targeting fungal pathogens. While promising, these approaches are still in the preclinical or early clinical stages.

Hematopoietic stem cell transplantation (HSCT) has been established as a curative therapeutic approach for certain IEIs, most notably SCID, where early transplantation significantly improves survival outcomes.

## 8. Implications for Practice

### 8.1. Integrating Preventive Strategies

The findings suggest that healthcare providers should incorporate preventive measures into routine prenatal care to reduce the risk of candidiasis in newborns.

### 8.2. Enhanced Patient Education

Educating parents about hygiene practices, breastfeeding benefits, and recognizing early signs of candidiasis can empower them to take proactive steps in managing their newborn’s health.

### 8.3. Interdisciplinary Collaboration

Encouraging collaboration among obstetricians, pediatricians, and neonatal care providers can lead to a more comprehensive approach to addressing and preventing candidiasis.

### 8.4. Regular Monitoring and Follow‐Up

Establishing protocols for regular monitoring of at‐risk newborns can help in the early identification and treatment of candidiasis, improving overall outcomes.

### 8.5. Ongoing Research and Training

The importance of continued research and training for healthcare professionals on best practices in managing and preventing candidiasis is emphasized, ensuring that they remain informed about the latest strategies.

## 9. Conclusion

Although significant progress has been made in the treatment of neonatal IC, substantial gaps remain, particularly concerning CNS infections and immunocompromised hosts. By including genetic testing and a clear clinical algorithm, healthcare providers can better manage neonatal candidiasis, particularly when IEI are suspected. Understanding the genetic basis of these defects will improve early diagnosis, enabling more targeted treatments and better clinical outcomes for neonates with severe or recurrent candidiasis. Future research must focus on optimizing antifungal dosing regimens in neonates, establishing the role of immune‐based therapies, and conducting prospective clinical trials tailored to this vulnerable population.

## Author Contributions

Study concept and design: Zahra Pourpak and Mahsa Fattahi. Acquisition of data and drafting of the manuscript: Mahsa Fattahi, Pegah Tamimi, and Aliasghar Ghaderi. Critical scientific revision of the manuscript: Mohammad Reza Fazlollahi and Zahra Pourpak.

## Funding

The study is funded by the Immunology, Asthma and Allergy Research Institute, Tehran University of Medical Sciences for providing this research opportunity (Grant 70353).

## Conflicts of Interest

The authors declare no conflicts of interest.

## Data Availability

The data used to support the findings of this study are available from the corresponding author upon request.
